# TRIB3 Promotes the Malignant Progression of Bladder Cancer: An Integrated Analysis of Bioinformatics and *in vitro* Experiments

**DOI:** 10.3389/fgene.2021.649208

**Published:** 2021-03-26

**Authors:** Jieping Yang, Jiaxing Lin, Jun An, Yongkang Zhao, Siyang Jing, Meng Yu, Yuyan Zhu, Yang Yao

**Affiliations:** ^1^Department of Urology, The First Hospital of China Medical University, Shenyang, China; ^2^Department of Urology, Shenzhen Second People’s Hospital, Shenzhen, China; ^3^Joint Laboratory of Artificial Intelligence and Precision Medicine of China Medical University and Northeastern University, Northeastern University, Shenyang, China; ^4^Department of Laboratory Animal Science, China Medical University, Shenyang, China; ^5^Department of Physiology, Shenyang Medical College, Shenyang, China

**Keywords:** TRIB3, bladder cancer, biomarker, proliferate, metastasis

## Abstract

**Background:**

Bladder cancer is a common malignant tumor characterized by high mortality and high management costs; however, it lacks useful molecular prognostic markers. Tribbles pseudokinase 3 (TRIB3) is a pseudokinase that participates in cell tumor progression and metabolism and whose function in bladder cancer is not precisely known.

**Main Methods:**

We downloaded transcriptome data and clinical data of bladder cancer from associated databases and extracted the expression matrix of TRIB3 for multiple bioinformatics analysis. RT-PCR detected the expression of TRIB3 in bladder cancer cells. After knockdown of TRIB3 with siRNA, we investigated TRIB3 function using CCK8, Cell Cycle and Transwell assays.

**Key Findings:**

Kaplan–Meier analysis of TRIB3 in the four cohorts showed that high expression of TRIB3 correlated with poor outcome. Expression of TRIB3 positively correlated with stage and grade and down-regulation of TRIB3 expression significantly inhibited proliferation, migration and cell cycle of bladder cancer cells.

**Significance:**

TRIB3 is a potential prognostic marker and therapeutic target. It can be used to individualize the treatment of bladder cancer.

## Introduction

Bladder cancer is common cancer worldwide. It is more common in men than in women, and its prevalence increases sharply with age (Yee et al., 2011). Treatment depends on the stage (Botteman et al., 2003; Steven and Poulsen, 2007), however, treatment responses and overall outcomes in patients at similar stages vary (Ye et al., 2014). The tumor tends to recur and metastasize (Holmäng et al., 1995; Steven and Poulsen, 2007). Therefore, there is an urgent need to identify molecular markers to predict treatment responses and to design individualized treatment (Ye et al., 2014).

Tribbles pseudokinase 3 (TRIB3) is a member of the pseudokinase gene family (Grosshans and Wieschaus, 2000). It lacks kinase activity due to a lack of ATP-specific binding sites and catalytic cores (Manning et al., 2002); however, it can compete with kinases for peptide substrates to regulate biological function (Qi et al., 2006). TRIB3 regulates a series of physiological processes, including cell proliferation, differentiation, apoptosis, glucose metabolism, lipid metabolism, and cell stress (Yokoyama and Nakamura, 2011). TRIB3 protein is also involved in signal pathways such as the MAPK pathway (Kiss-Toth et al., 2004), the PI3K pathway (Ding et al., 2008), the NF-κB pathway (Wu et al., 2003), and TGF-beta (Hua et al., 2011). These findings suggest that TRIB3 participates in various physiological and pathological processes. The expression of TRIB3 is higher in breast cancer (Wennemers et al., 2011), ovarian cancer (Wang et al., 2020), lung cancer (Zhang X. et al., 2019), and colorectal cancer (Hua et al., 2019), and outcomes are poor. However, few studies reported the role of TRIB3 expression in bladder cancer.

In the present study, we used several bladder cancer cohorts and online databases to explore the prognostic potential of TRIB3 and to explore the mechanisms of TRIB3 in bladder cancer cells through functional experiments *in vitro*.

## Materials and Methods

### Downloading and Filtering Raw Data

The Cancer Genome Atlas (TCGA^1^) is the most authoritative oncology database. We downloaded the transcriptome and sample information, Gene Ontology Consortium (GEO^2^) and ArrayExpress from TCGA.^3^ also contain substantial amounts of sequencing data. We obtained transcriptome and sample information from these two databases for GSE32548 (*n* = 146) (Lindgren et al., 2012), GSE32894 (*n* = 308) (Sjödahl et al., 2012) and E-MTAB-1803 (*n* = 85) (Rebouissou et al., 2014). In the R language environment, we standardized all RNA expression matrices and calculated log values using the R package “edge.” Finally, we selected patients with complete transcriptional sequencing and follow-up information. The final numbers of cases included in the analyses were 403 (TCGA-BLCA), 130 (GSE32548), 224 (GSE32894), and 73 (E-MTAB-1803). Specific clinical information is displayed in Table 1.

**TABLE 1:** Summary of basic clinical information of four bladder cancer cohorts.

Clinical Factors	TCGA_BLCA		E-MTAB-1803		GSE32548		GSE32894	
				
	*n* = 403	%	*n* = 73	%	*n* = 130	%	*n* = 224	%
**Age**								
≤60	107	26.55	22	30.13	27	20.77	46	20.54
>60	296	73.45	51	69.86	103	79.23	178	79.46
**Gender**								
Male	298	73.95	62	84.93	99	76.15	163	72.77
Female	105	26.05	11	15.07	31	23.85	61	27.23
**T stage**								
>T2	4	0.99	0	0	91	70	173	77.23
≥T2	366	94.29	73	100	38	29.23	51	22.77
**Grade (WHO2004)**								
Low	20	4.96	–	–	–	–	–	–
High	380	94.29	–	–	–	–	–	–
**Grade (WHO1973)**								
G1	–	–	0	0	15	11.54	45	20.09
G2	–	–	4	5.48	40	30.77	84	37.50
G3	–	–	69	94.52	75	57.69	93	41.52
**Vital status**								
Alive	248	61.54	30	41.10	105	80.77	199	88.84
Dead	155	38.46	43	58.90	25	19.23	25	11.16
**Follow-up (mean ± SD, year)**								
	2.10 ± 2.23		2.40 ± 2.44		4.14 ± 2.38		3.28 ± 2.10	

### Kaplan–Meier Analysis

We used the R packages “survival” and “survminer” for survival analysis. We calculated expression values using the function “res. cat.” We used the best cut-off value to divide patients into high-risk and low-risk groups. We performed Kaplan–Meier analysis on the two groups when *p* < 0.05, using the online tool OSblca^4^ (Zhang G. et al., 2019). The database integrates multiple bladder cancer data sets and is a powerful tool for evaluating bladder cancer prognostic markers. We downloaded transcriptome and survival data containing 195 bladder cancer samples from the attachment to the article for survival validation (Mariathasan et al., 2018).

### Univariate and Multivariate Cox Regression Analysis

We used R-package “survival” for univariate and multivariate Cox regression analysis. Multivariate Cox analysis included age, sex, primary tumor extent (T stage), grade and prognostic factor gene.

### Receiver Operating Characteristic (ROC) Curve

We plotted the ROC curve of overall survival using the R package “survivalROC,” and calculated the area under the curve (AUC). AUC > 0.5 generally indicates specific prediction ability. Higher AUC suggests more accurate prediction results of the model.

### Concordance Index (C-Index)

C-index is used to calculate the discrimination between the predicted and real values of the model in survival analysis. We use R package “survcomp” and “survival” to fit multiple factors for survival analysis and calculate the c-index.

### UALCAN Database

UALCAN is a comprehensive and interactive internet resource for analyzing cancer OMICS data^5^ (Chandrashekar et al., 2017). Using this tool, one can query the mRNA distribution of genes in normal and tumor tissues.

### Metabase Enrichment Analysis

The internet-based tool cBioPortal queries the co-expression genes of target genes. Spearman coefficients greater than 0.3 indicate that there is co-expression. We analyzed these genes using pathway and process enrichment analysis of the Metabase database^6^ (Zhou et al., 2019). The enrichment analysis included KEGG Pathway, GO Biological Processes, Reactome Gene Sets, Canonical Pathways, and CORUM to evaluate these genes’ potential biological functions and pathways. We selected term enrichment significance according to *p*-value < 0.01, minimum count of 3, and enrichment factor >1.5.

### BioGRID and STRING Database

BioGRID^7^ is a biomedical interaction repository. The database can be used for searching publications for protein and genetic interactions, chemical interactions, and post-translational modifications from significant model organism species. String^8^ database is used for searches of known protein-protein interactions, and it predicts protein-protein interactions.

### Human Samples and Ethics Statements

A total of 40 pairs of bladder cancer tissues and para-cancer tissues were collected in the Department of Urology, the First Hospital of China Medical University (Shenyang). The age range of these patients is 38–80 years. The protein extracted from 40 pairs of samples was analyzed by western blot to detect the difference of TRIB3 expression between cancer tissues and para-cancer tissues. The Ethics Committee of the First Hospital of China Medical University approved this study according to the Declaration of Helsinki, and written informed consent was obtained from all the patients.

### Cell Culture and Transfection

We cultured T24 and UMUC3 cells in DMEM (Gibco, United States) containing 10% FBS (Gibco) at 37°C and saturated humidity with 5% CO_2_. The day before transfection, we seeded exponentially growing cells onto 6-well plates. To increase the transfection efficiency, we added serum-free medium to each well before transfection. We diluted 6 μl Lipofectamine RNAiMAX (Invitrogen, Carlsbad, CA, United States) with 125 μl Opti-MEM (Gibco) and added diluted 3 μl TRIB3-siRNA or TRIB3-nc (RiboBio Co., Ltd., CHINA) with 125 μl Opti-MEM (Gibco). We mixed for 5 min and added them to 6-well plates. We incubated the cells at 37°C in 5% CO_2_ was incubated for 12 h. We then replaced the medium with DMEM containing 10% FBS. We performed CCK8 and Transwell assays 24 h after transfection and extracted RNA 48 h after transfection. The TRIB3-siRNA sequences were genOFFTM st-h-TRIB3_001: GGAGTTGGATGACAACTTA and genOFFTM st-h-TRIB3_002: CTACGTGGGACCTGAGATA.

### Western Blot Experiment

RIPA buffer (Sigma, St. Louis, MO, United States) was added to the paracancerous-tissue and cancerous-tissue, which was lysed by sonication for 30 min, collecting the tissue lysates at 13,000 rpm for 30 min, at 4°C. And each protein concentration was calculated by the BCA method (Solarbio, Beijing, China). Protein (40 μg) were separated by 10% SDS-PAGE and transferred onto PVDF membranes (Merck Millipore, Billerica, MA, United States), and then blocked by 5% skim milk the membranes were incubated with the dilution (1:1,000) of specific primary antibodies at 4°C overnight. TRIB3 (ImmunoWay Biotechnology, United States, YN1887), β-Actin (Bioss Antibodies, United States) Then, the membranes were incubated with the secondary antibodies for 2 h at room temperature. Goat anti-rabbit IgG (Santa Cruz Biotechnology, United States). the immune-reactive proteins were visualized using Pierce^TM^ ECL Plus western blotting substrate (Thermo Fisher Scientific).

### RT-PCR

We extracted total RNA using TRIzol^®^ reagent (Thermo Fisher Scientific) according to the manufacturer’s protocols and determined the purity and concentration of RNA solution using a NanoDrop 2000 spectrophotometer (NanoDrop Technologies; Thermo Fisher Scientific). We used the RT-PCR kit (Takara, China) of SuperScript III First-Strand Synthesis System to reverse transcribe RNA into cDNA. We performed RT–PCR using the SYBR premix ExTaq^TM^ kit (Takara, China). The amplification conditions were as follows: denaturation at 95°C for 30 s, 95°C for 5 s, and 60°C for 30 s, for a total of 40 cycles. β-actin primer: Forward: 5′- CAGCATTGGCAATGAGGGGTTC-3′, Reverse: 5′- AGGTCTTTGCGGATGTCCACGT-3′. TRIB3 primer: Forward: 5′- TGCCCTACAGGCACTGAGTA-3′, Reverse: 5′-GTCCGAGTGAAAAAGGCGTA-3′. We performed quantitative real-time PCR amplification using the LightCycler^TM^480 II system (Roche Diagnostics, Switzerland) and calculated results using the 2^–ΔΔCT^ method.

### Cell Proliferation Assay

We prepared exponentially growing cells as single-cell suspensions. We seeded cells in 96-well plates (2,000 cells/well) and assessed them at 0, 24, 48, 72, and 96 h. Time 0 indicated 6 h post-plating. Following the addition of 10 μl of CCK8 solution (Vazyme, Nanjing, China) to each well, we incubated cells for 3 h at 37°C in a 5% CO_2_ atmosphere. We incubated the plate with shaking for 5 min. We measured optical density (OD) at 490 nm, using a Microplate reader (Bio-Rad, CA, United States). We performed these experiments in triplicate.

### Transwell Assay

We used the Transwell assay to determine the effect of TRIB3 on the migration abilities of T24 and UMUC3 cells. Twenty-four hours after transfection, we collected cells and assayed them. We digested cells and prepared cell suspension. We added 600 μl of medium containing 10% FBS to the lower chamber and added 200 μl of the cell suspension (1 × 10^5^ cells/ml) in serum-free medium to the upper chamber. We incubated the Transwell at 37°C for 24 h. We removed the medium from the upper chamber and fixed the cells with 4% paraformaldehyde for 30 min. Then, we washed the cells three times with PBS and stained them with 0.2% crystal violet for 30 min. Finally, we photographed and counted the cells in five independent microscopic fields. We performed these experiments in triplicate.

### Cell Cycle Assay

The bladder cancer cells transfected by TRIB3-NC and TRIB3-siRNA for 24 h were collected, and digested with trypsin, and washed three times with PBS, and centrifuged at 2000 *g* for 5 min, then removing the supernatant. Adjust the cell concentration to 2 × 106 cells/ml. A 700 μl of 100% chilled ethanol and 300 μl pre-cool PBS was added to the cells, which was fixed for 2 h at −20°C. And centrifuged at 2000 *g* for 5 min and removed the ethanol. 500 μl of the PI/RNase (Solarbio, Beijing, China) staining working solution (PI:RNase A was prepared at 4:1) was added. Following staining at 37°C for 30 min, flow cytometry was performed.

## Results

### TRIB3 Predicts Overall Survival in Bladder Cancer Cohorts

**FIGURE 1:**
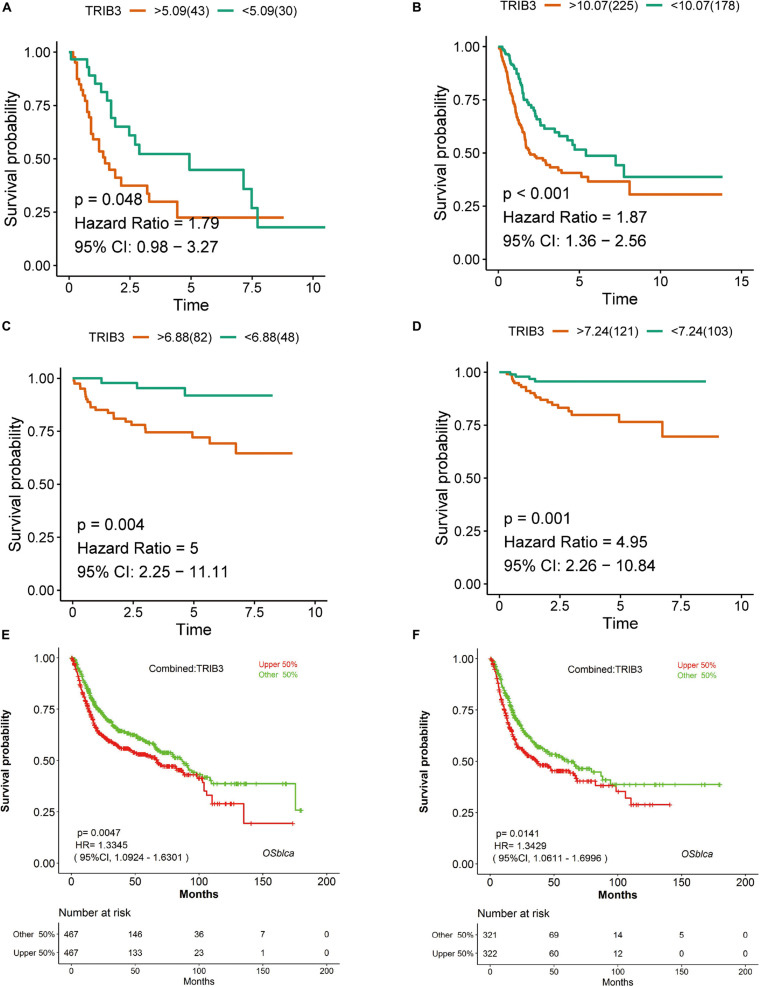
Kaplan–Meier analysis of TRIB3 expression. **(A)** E-MTAB-1803. **(B)** TCGA_BLCA. **(C)** GSE32548. **(D)** GSE32894. **(E)** Kaplan–Meier curve of TRIB3 expression in a meta-cohort of bladder cancer. **(F)** Kaplan–Meier curve of TRIB3 expression in a meta-cohort of muscle-invasive bladder cancer. Red represents the TRIB3 high expression group, green represents the TRIB3 low expression group, and CI represents the confidence interval.

In four cohorts, we divided samples into two groups according to the expression of TRIB3 for Kaplan–Meier analysis. There were significant differences in E-MTAB-1803 (*p* = 0.048) (Figure 1A), TCGA_BLCA (*p* < 0.001) (Figure 1B), GSE32548 (*p* = 0.004) (Figure 1C) and GSE32894 (*p* = 0.001) (Figure 1D). The outcomes of the high-expression groups in all four cohorts were worse than those of the low-expression group. We verified the ability of TRIB3 to predict overall survival using OSblca. The tool integrated the data of TCGA-BLCA, GSE13507, GSE31684, GSE48075, and GSE48276 cohorts (*n* = 934) to draw Kaplan-Meier overall survival curves for total bladder cancer (Figure 1E) and muscle-invasive bladder cancer (Figure 1F). Outcomes in the high expression TRIB3 group were poor (*p* < 0.05).

### ROC Curve and AUC Value of TRIB3 on Overall Survival Rate

**FIGURE 2:**
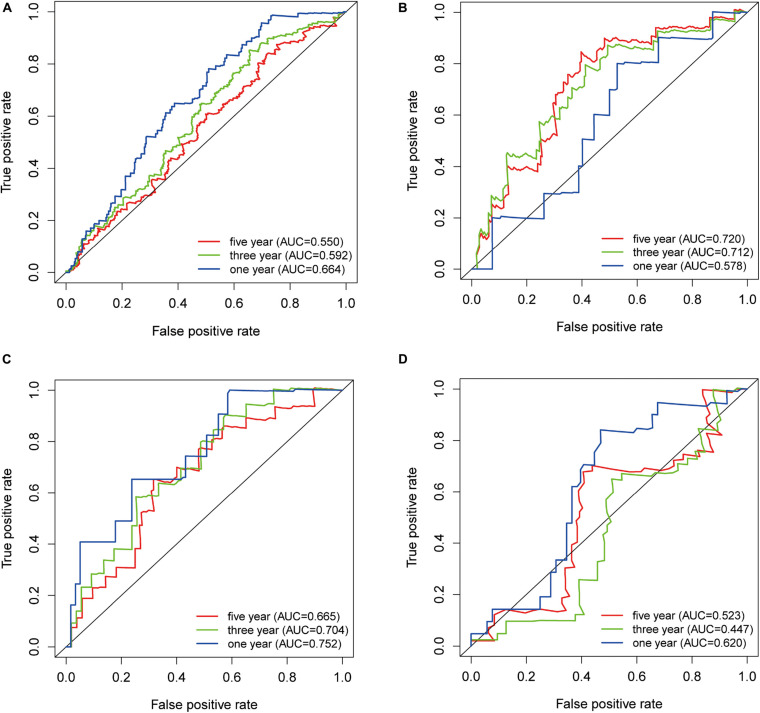
ROC curve. **(A)** TCGA-BLCA. **(B)** GSE32894. **(C)** GSE32548. **(D)** E-MTAB-1803. The blue, green, and red lines represent the ROC curve of 1-year, 3-year, and 5-year overall survival rates.

Receiver operating characteristic curve and AUC values return diagnostic values of markers. We drew 1-, 3-, and 5-year ROC curves of TRIB3 expression on the overall survival rate in four cohorts and calculated the AUC corresponding to each curve. The AUCs of the 1-, 3-, and 5-year TCGA-BLCA cohorts were 0.664, 0.592, and 0.550, respectively (Figure 2A). The AUCs of the 1-, 3-, and 5-year GSE32894 cohorts were 0.578, 0.712, and 0.720, respectively (Figure 2B). The AUC values of the 1-, 3-, and 5-year GSE32548 cohorts were 0.752, 0.704, and 0.665, respectively (Figure 2C). The AUC values of the 1-, 3-, and 5-year E-MTAB-1803 cohorts were 0.620, 0.447, and 0.523 (Figure 2D).

### Univariate and Multivariate COX Analysis

We performed univariate and multivariate analysis of clinical factors (Age, sex, T stage, grade) and TRIB3 (Table 2). Univariate COX analysis showed that TRIB3 and T-stage had prognostic ability in all four cohorts. Multivariate COX analysis showed that T stage still had independent prognostic ability in four cohorts, while TRIB3 had independent prognostic ability in cohort TCGA-BLCA, GSE32548, and GSE32894. To sum up, the prognostic ability of TRIB3 is stronger than that of age, sex, and grade. Than we calculated the C-index of TRIB3, T stage, and integrative model (Combination of TRIB3 and T stage). The C-index of them are all more 0.6 in the four cohort. However, the C-index of T stage is more than the C-index of TRIB3. In the TCGA-BLCA and GSE32894 cohorts, the C-index of integrative model is more than T stage, and it’s the opposite in the GSE32548 and E-MTAB-1803 cohorts. Therefore, whether we can combine TRIB3 and T stage for survival prediction needs a larger cohort to explore (Supplementary Table 1).

**TABLE 2:** Univariate and multivariate Cox regression analysis of clinical-factors/TRIB3 with overall survival rate in patients.

Variables	Univariate analysis		Multivariate analysis	
		
	HR (95% CI)	P	HR (95% CI)	P
**TCGA-BLCA**				
TRIB3	1.24 (1.1–1.4)	6.00E-04	1.2 (1.05–1.36)	6.98E-03
Age	2.29 (1.48–3.54)	1.87E-04	2.06 (1.28–3.31)	3.03E-03
Gender	0.9 (0.63–1.28)	5.56E-01	0.79 (0.55–1.14)	2.02E-01
Grade	9608547.15 (0-Inf)	9.91E-01	3911230.59 (0.00–Inf)	9.92E-01
T stage	1.76 (1.37–2.24)	6.67E-06	1.74 (1.34–2.24)	2.58E-05
**GSE32548**				
TRIB3	1.77 (1.21–2.59)	3.40E-03	1.71 (1.02–2.85)	4.12E-02
Age	2.18 (0.65–7.28)	2.07E-01	2.17 (0.63–7.43)	2.19E-01
Gender	1.27 (0.48–3.39)	6.30E-01	1.51 (0.55–4.21)	4.25E-01
Grade	2.29 (1.08–4.86)	3.02E-02	0.54 (0.18–1.58)	2.58E-01
T stage	3.53 (1.89–6.6)	7.42E-05	4.54 (1.86–11.12)	9.20E-04
**GSE32894**				
TRIB3	1.73 (1.14–2.61)	9.76E-03	1.46 (1.02–2.08)	3.87E-02
Age	0.72 (0.3–1.72)	4.54E-01	1.08 (0.41–2.82)	8.81E-01
Gender	1.54 (0.58–4.1)	3.89E-01	0.96 (0.35–2.64)	9.36E-01
Grade	7.59 (2.45–23.52)	4.45E-04	1.66 (0.46–6.03)	4.39E-01
T stage	4.74 (2.98-7.54)	5.16E-11	4.13 (2.28–7.48)	2.95E-06
**E-MTAB-1803**				
TRIB3	1.27 (1.15–1.47)	9.00E-03	1.21 (0.74–1.97)	4.53E-01
Age	1.23 (0.64–2.36)	5.37E-01	1.11 (0.54–2.29)	7.79E-01
Gender	1.08 (0.45–2.59)	8.63E-01	1.39 (0.56–3.44)	4.74E-01
Grade	0.88 (0.31–2.49)	8.12E-01	0.88 (0.27–2.83)	8.32E-01
T stage	2.92 (1.88–4.53)	1.74E-06	3.05 (1.92–4.82)	2.00E-06

*HR, hazard ratio; CI, confidence interval; Inf, infinity; Bold font means statistically significant; Inf, Infinity.*

### TRIB3 Is Positively Correlated With the Grade of Bladder Cancer

**FIGURE 3:**
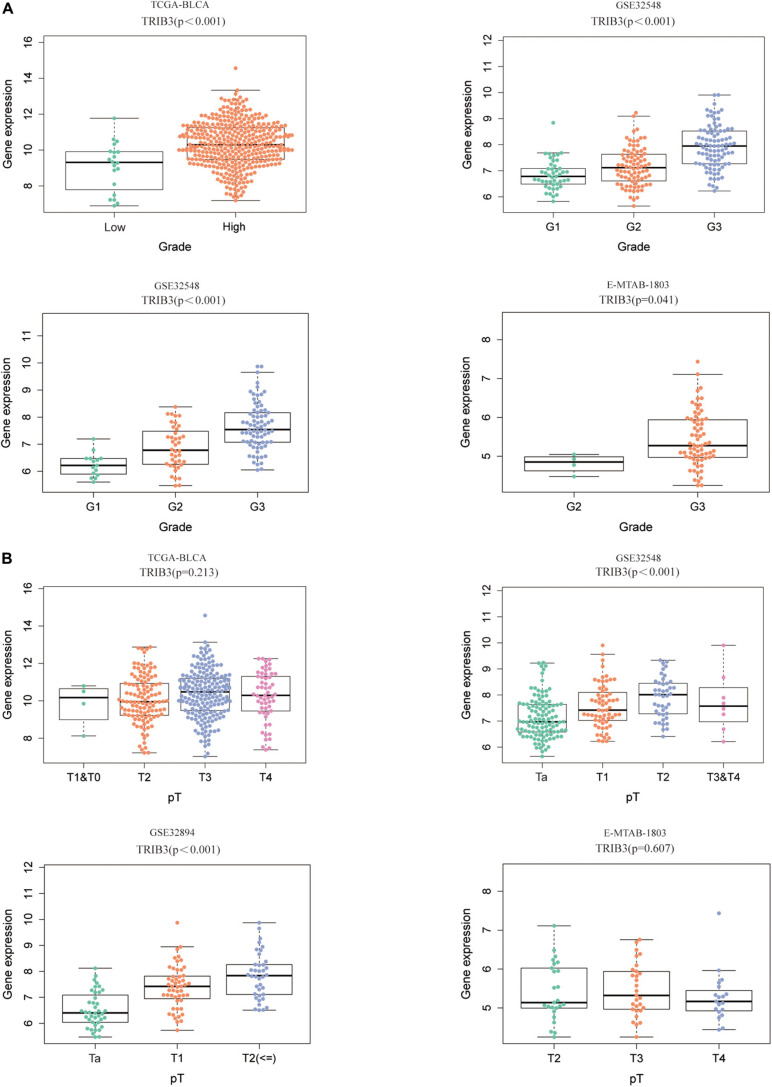
The expression of TRIB3 in various clinical types. **(A)** The expression scatters the plot of TRIB3 under different grades in four cohorts. **(B)** The expression scatters the plot of TRIB3 under different T stages in four cohorts.

We compared the difference of TRIB3 expression in various grades and T stages. We found that expression levels of TRIB3 differed according to grades and T stage of TCGA-BLCA, GSE32894, GSE32548, and E-MTAB-1803 (*p* < 0.05, Figures 3A,B). High grade correlated with a trend toward increased expression levels of TRIB3. In cohort GSE32548 and GSE32894, the expression of TRIB3 was different only in different grades and T stages (*p* < 0.05, Figures 3A,B).

### There Is Differential Expression of TRIB3 in Various Types of Bladder Cancer

**FIGURE 4:**
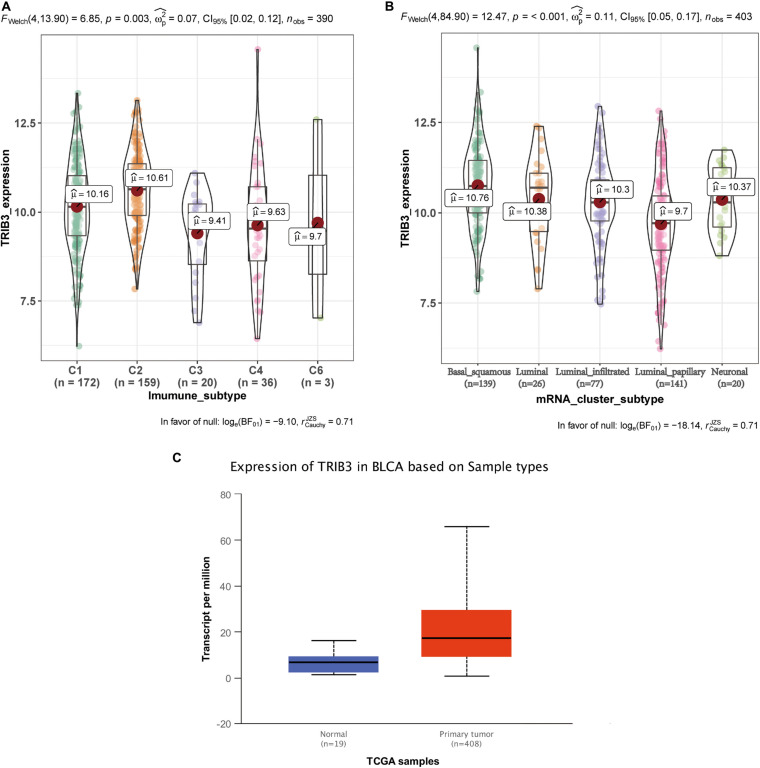
The differential expression of TRIB3 in other typing schemes. **(A)** Expression levels of the five immunophenotypes: C1 (wound healing), C2 (IFN-gamma dominant), C3 (inflammatory), C4 (lymphocyte depleted), and C6 (TGF-b dominant). **(B)** Expression levels in five mRNA types: basal squamous, luminal, luminal infiltrated, luminal papillary, and neuronal subtype. **(C)** Comparison of expression levels of TRIB3 in normal tissue and tumor tissue. *p* < 0.001 indicates statistical significance.

We obtained data on bladder cancer typing from UCSC Xena^9^. The expression of TRIB3 in various immune subtypes also differed (*p* < 0.05) (Figure 4A). Among them, the expression level of TRIB3 of basal squamous subtype is the highest. The expression of TRIB3 differed depending on the mRNA cluster (*p* < 0.001, Figure 4B). Among them, the expression level of TRIB3 of C2 (IFN-gamma dominant) is the highest. Using web tool “UALCAN” to query the data of TRIB3 expression in bladder cancer tissues and normal tissues, we found that TRIB3 was highly expressed in tumor tissues (*p* < 0.001, Figure 4C).

### TRIB3 Expression Level in Bladder Cancer Tissues and Its Clinical Significance

**FIGURE 5:**
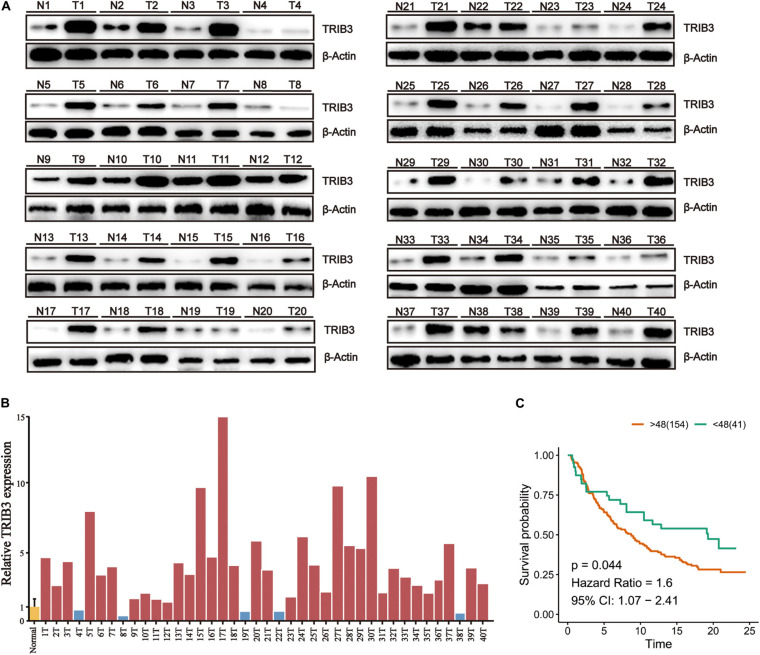
High TRIB3 expression in bladder cancer tissues of patients. **(A)** The expression of TRIB3 protein in bladder cancer tissues and paracancer tissues by western blot **(B)** The relative gray values of TRIB3 protein in bladder cancer tissues. **(C)** Kaplan–Meier analysis of TRIB3 expression in validation cohort.

To confirm the above results, we performed western blot experiment on tissues samples obtained from 40 bladder cancer patients operated at the First Hospital of China Medical University. The results show that the expression of TRIB3 in cancer tissues was significantly higher than the paracancer tissues (Figure 5A), and confirmed by Image J (NIH, United States) which was used to densitometer quantitative analysis of TRIB3 Western blot results of 40 pairs of tissues (Figure 5B). The analysis results showed that TRIB3 was relatively highly expressed in 35 of the 40 pairs of bladder cancer tissue samples (Figures 5A,B). Moreover, to assess the clinical significance of TRIB3 expressions in bladder cancer, the clinical characteristics between TRIB3 and bladder cancer were evaluated in tissues. It was revealed that high expression levels of TRIB3 were significantly associated with higher tumor TNM stage (Table 3). However, there are no significant statistical significance between the expression of trib3 and whether distant metastasis occurs. We believe it is due to the insufficient number of bladder cancer patients with metastasis. Taken together, our observations strongly suggest that TRIB3 might have a pro-oncogenic role in bladder cancer. Finally, we used Sanjeev’s bladder cancer cohort for verification, and it was found that TRIB3 still had the ability of prognosis (*p* < 0.05, Figure 5C).

**TABLE 3:** The clinical significance of TRIB3 expressions in bladder cancer.

Characteristic	TRIB3 expression		Sum (40)	P*value	
			
	<median (*n* = 20)	≥median (*n* = 20)		Chi-square test	Fisher exact test
**Age**				0.5186	0.7475
<60	9	7	16		
≥60	11	13	24		
**Gender**				0.1675	0.3008
Male	12	16	28		
Female	8	4	12		
**TNM stage**				0.0181*	0.0407*
I-II	17	10	27		
III-IV	3	10	13		
**Distant metastasis**				0.0765	0.1818
Negative	19	15	34		
Positive	1	5	6		

* *p* < 0.05 was considered significant. Bold values means statistically significant.

### Down-Regulation of TRIB3 Induces Cell Cycle Arrest in G0/G1 Phase

**FIGURE 6:**
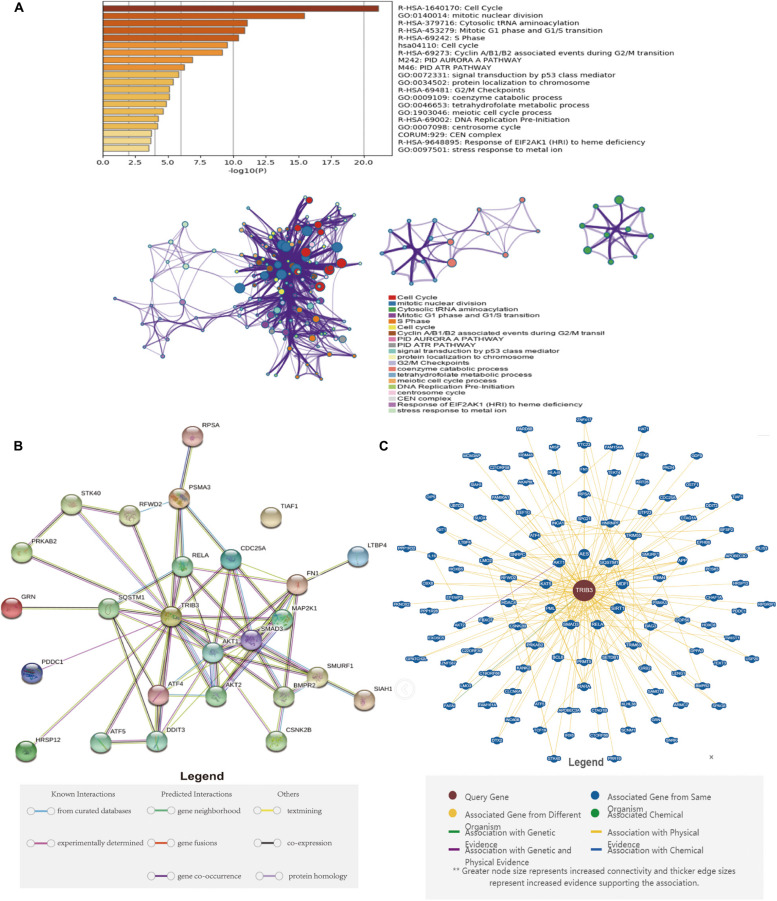
Metascape enrichment analysis and protein interactions. **(A)** The Metascape enrichment analysis results show that larger log10 (P) correlates with the significance of the enrichment. **(B)** Protein-protein interactions in the STRING database. **(C)** Protein-protein interactions in the BioGRID database.

**FIGURE 7:**
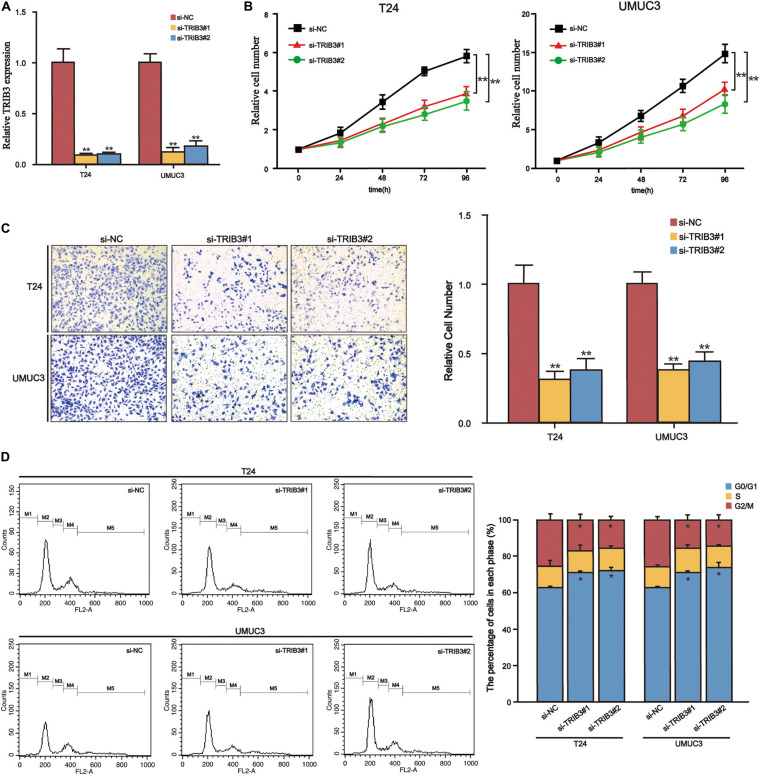
TRIB3-siRNA inhibits the proliferation and migration and induces cell arrest in bladder cancer cells. **(A)** Relative mRNA levels of control and si-TRIB3 in T24 and UMUC3 cells. **(B)** The CCK-8 assay results showed that T24 and UMUC3 cells’ proliferation ability decreased when treated with si-TRIB3. **(C)** The invasion assay shows that the invasive ability of T24 and UMUC3 cells treated with si-TRIB3 decreased. **(D)** G0/G1 phase arrest of bladder cancer after TRIB3 siRNA transfection, confirmed by statistical analysis. *P < 0.05 and **P < 0.01.

We searched for genes that positively correlate to TRIB3 expression in the BLCA (TCGA 2017) cohort of the cBioPortal website (Spearman’s Correlation >2017). We introduced these genes into the Metascape database for enrichment analysis. The three items with significant enrichment were Cell Cycle, mitotic nuclear division, and Cytosolic tRNA aminoacylation (Figure 6A), all of which relate to the cell cycle. Then flow cytometry verified that the proportion of the bladder cancer cells transfected by TRIB3-siRNA in the G0/G1 phase was significantly increased (*p* < 0.05), as compared to the cells transfected by TRIB3-NC (Figure 7D).

### Proteins Interacting With TRIB3

We found that TRIB3 and AKT1/AKT2 are the most closely related (Figure 6B). The BioGRID database showed Physical Edges between TRIB3 and a large number of proteins (Figure 6C). The most significant correlations were AES, SQSTM1, MDF1, SIRT1, RELA, SMAD3, PML, KAT5, and AKT1.

### Further Study of TRIB3 in a Bladder Cancer Cell Knockdown Test

Because of the high expression of TRIB3 in bladder cancer and poor outcome, we knocked down TRIB3 in bladder cancer cells and conducted functional experiments further to explore the function of TRIB3 (Figure 7A). CCK8 analysis showed that the proliferation ability of si-TRIB3 cells was significantly lower (*p* < 0.05) (Figure 7B). The Transwell analysis showed that si-TRIB3 bladder cancer cells’ migration ability was significantly lower (*p* < 0.05) (Figure 7C).

## Discussion

Bladder cancer is one of the most common malignant tumors worldwide. Because of the lack of specific characteristics in the early stages, most patients present with advanced bladder cancer. Bladder cancer treatment includes surgery, chemotherapy, radiotherapy, and poor outcomes (Marta et al., 2012; Racioppi et al., 2012). Mining robust molecular markers and therapeutic targets stratify patients at risk to facilitate treatment decision-making and individualized treatment. In the present study, we combined computational biology methods and experimental verification methods to identify and characterize the role of pseudokinase-TRIB3 in poor outcomes and malignant progression of bladder cancer. We demonstrated the utility of integrating bioinformatics and *in vitro* experiments to explore potential diagnostic and therapeutic targets for bladder cancer.

One of the main findings of this study was that TRIB3 is a robust prognostic biomarker of bladder cancer. We obtained datasets from TCGA-BLCA, GSE32548, GSE32894, and E-MTAB-18034. Kaplan–Meier analysis of all four cohorts showed that high expression levels of TRIB3 correlated with shorter survival rates. The AUC values of 1BI 3 and 5 years in the cohort were almost all greater than 0.5, suggesting that TRIB3 expression levels can predict risk at these time points. TRIB3 successfully predicted outcome in patients with bladder cancer in two meta-cohorts. These results suggest that expression levels of TRIB3 may serve as an index to predict outcomes for patients with bladder cancer. The four queues used in this study derive from GEO, TCGA, and ArrayExpress databases. These three databases are currently the largest three transcriptome databases. Data upload has strict requirements and high reliability. The four queues’ samples derive from several regions, which suggests that TRIB3 expression a wide range of generalizability. Our study’s scale was polycentric, cross-regional, and large-scale, which increases the reliability of our results.

Another important finding was that TRIB3 is a potential oncogene in bladder cancer. We identified TRIB3 expression in all four cohorts. Expression levels of TRIB3 increased with increased stage and grade, suggesting that TRIB3 promotes the progression of bladder cancer and increases the risk of invasion and deterioration of bladder cancer. We confirmed high expression levels of TRIB3 mRNA in bladder cancer cells using qRT-PCR. After inhibiting the expression of TRIB3 in bladder cancer cells, we found that the proliferation and migration of bladder cancer cells decreased. This experiment suggests that expression of TRIB3 plays a tumor-promoting role in bladder cancer. Studies reported that TRIB3 promotes acute promyelocytic leukemia by stabilizing oncoprotein PML-RAR α and inhibiting p53-mediated aging (Li K. et al., 2017). Knockout of TRIB3 inhibits tumorigenesis and cancer progression. It disrupts the TRIB3-SQSTM1 or TRIB3-PML/RAR α interaction through specific peptides, and this is a potential strategy for treating certain solid cancers and acute promyelocytic leukemia (Hua et al., 2011; Li K. et al., 2017). Studies confirmed that inhibition of the expression of TRIB3 in ovarian cancer cells causes cell cycle arrest in the G0/G1 phase in ovarian cancer cells. Proliferation, migration, and invasion of ovarian cancer cells reduce, and apoptosis increases (Wang et al., 2020). Enrichment analysis of co-expressed genes of TRIB3 showed that TRIB3 was related to the cell cycle (Figure 6A). Flow cytometry analysis revealed that arrested G0/G1 cell cycle could be induced by TRIB3-siRNA, confirmed by statistical analysis (Figure 7D), thereby inhibiting the proliferation and migration of bladder cancer cells. Other studies confirmed that the expression of TRIB3 is greater in solid tumors such as colorectal cancer, breast cancer, lung cancer, ovarian cancer, melanoma, liver cancer, and leukemia (Wennemers et al., 2011; Salazar et al., 2015; Hua et al., 2019; Zhang X. et al., 2019; Wang et al., 2020; Zhu et al., 2020), suggesting that TRIB3 is a potential target for cancer treatment. Some studies have shown that biological scaffold coordination complexes can be used to design new target-specific therapeutic agents, and TRIB3 is also a scaffold protein that acts as a platform response. In the future, we can try to combine TRIB3 with new compounds for combined therapy (Mirgayazova et al., 2019). We conclude from these results that TRIB3 is a potential oncogene and a potential therapeutic target for bladder cancer.

## Conclusion

Overall, TRIB3 successfully predicted outcomes in patients with bladder cancer in four cohorts. It may serve as a prognostic marker for individualized treatment of bladder cancer in the future. TRIB3 expression promotes the proliferation and invasion of bladder cancer cells. Although more clinical samples and more in-depth experiments are needed to explore the mechanism of TRIB3 in the future, our results suggest that TRIB3 is a potential prognostic marker and therapeutic target in bladder cancer.

## Data Availability Statement

The original contributions presented in the study are included in the article/Supplementary Material, further inquiries can be directed to the corresponding authors.

## Author Contributions

JY and JL contributed to conception and design. JY, JL, and MY carried out analysis and wrote the manuscript. JA, YKZ, and SJ collected and processed the data. YYZ and YY edited the manuscript and provided constructive comments. All authors read and approved the final manuscript.

## Conflict of Interest

The authors declare that the research was conducted in the absence of any commercial or financial relationships that could be construed as a potential conflict of interest.

## Footnotes

1 https://cancergenome.nih.gov/

2 http://www.ncbi.nlm.nih.gov/geo/

3 https://www.ebi.ac.uk/arrayexpress/

4 http://bioinfo.henu.edu.cn/BLCA/BLCAList.jsp

5 http://ualcan.path.uab.edu/index.html

6 http://metascape.org

7 https://thebiogrid.org/

8 https://string-db.org/

9 http://xena.ucsc.edu/

## Supplementary Material

Supplementary Table 1 C-index of the TRIB3, T stage and Integrative model.

## Abbreviations

AUC: area under the curve
GEO: Gene Expression Omnibus
ROC: receiver operating characteristic
TCGA: The Cancer Genome Atlas.
