# Supplementary material for: TRIB3 Promotes the Malignant Progression of Bladder Cancer: An Integrated Analysis of Bioinformatics and in vitro Experiments
Source: Front Genet. 2021 Mar 26;12:649208. doi: 10.3389/fgene.2021.649208 (PMC8033215; doi:10.3389/fgene.2021.649208)
Supplement: Supplementary Table 1 — C-index of the TRIB3, T stage and Integrative model. [file Table_1.docx]

Supplement table 1. C-index of the TRIB3，T stage and Integrative model.

|  | TRIB3 | T stage | Integrative model |
| --- | --- | --- | --- |
| TCGA-BLCA | 0.62 | 0.67 | 0.69 |
| GSE32548 | 0.65 | 0.87 | 0.79 |
| GSE32894 | 0.68 | 0.79 | 0.83 |
| E-MTAB-1803 | 0.6 | 0.79 | 0.72 |
